# Surgical and oncologic approach to leiomyosarcoma of the inferior vena cava: A case report

**DOI:** 10.1002/ccr3.9336

**Published:** 2024-08-20

**Authors:** Feleke Hailmariam Maniso, Mathewos Assefa Woldegeorgis, Hawi Furgassa Bedada

**Affiliations:** ^1^ Department of Clinical Oncology Tikur Anbessa Specialized Hospital, College of Health Science, Addis Ababa University Addis Ababa Ethiopia; ^2^ Oncology Unit, St. Paul's Hospital Millennium Medical College Addis Ababa Ethiopia

**Keywords:** case report, Ethiopia, inferior vena cava, leiomyosarcoma

## Abstract

**Key Clinical Message:**

LMS of IVC needs a multidisciplinary approach. Surgical excision with free margin is the cornerstone of management. Upon case‐by‐case selection, adjuvant chemotherapy may play a role in better oncologic outcome.

**Abstract:**

Leiomyosarcoma (LMS) of the inferior vena cava (IVC) is a rare form of mesenchymal origin malignancy with less than 400 cases reported to date. Surgery is the mainstay of management but it requires vast experience in vascular and visceral surgery to attain a free tumor margin. Subsequent adjuvant treatment with chemotherapy and radiation remains as an area of gray zone. We report the case of a 61‐year‐old man with an 8‐month history of abdominal pain. Upon physical examination, an ill‐defined mass over the right side of the lower abdomen and bilateral lower extremity edema were detected. Abdominal ultrasound with Doppler revealed a right‐side retroperitoneal mass invading the IVC with extensive venous thrombosis for which anticoagulation was initiated. Computed Tomography of the abdomen revealed a huge heterogeneously enhancing mass involving the whole length of the infrarenal IVC obstructing the IVC lumen with collateral veins draining through the paralumbar veins and inferior epigastric veins bilaterally. With a top differential of primary IVC LMS, a midline longitudinal laparotomy was performed with an intraoperative finding of a tumor arising from the infra‐renal IVC which was excised. Gore‐Tex graft was used to reconstruct the IVC. There was an injury to the right common iliac artery and it was repaired by end‐to‐end anastomosis. Histopathology confirmed a high‐grade LMS of the IVC and surgical margin status was unknown. He was given adjuvant Chemotherapy consisting of Doxorubicin and Dacarbazine. He has been on follow‐up at the Oncology side with a good performance status.

## 1 INTRODUCTION

Vascular leiomyosarcoma (VLMS) is generally a rare type of Leiomyosarcoma (LMS) originating from the smooth muscle layer of the vessels.^1^ Compared to arterial LMS, venous LMS is observed more often.^2^ LMS of the inferior vena cava (IVC) accounts for 5% of all LMS and more than 50% of VLMS.^1^ IVC LMS is indeed an exceptionally rare malignancy with fewer than 400 cases reported.^3^

The primary treatment is complete surgical resection with a negative margin, and additional adjuvant therapy after surgery may improve the prognosis.^3^, ^4^ However, due to its rare occurrence, there is still no consensus on the most suitable surgical approach, and postoperative adjuvant therapy. This case report aims to add a body of knowledge and practical approach to the diagnostic and treatment strategies for LMS of the IVC in a resource‐limited setup.

## 2 CASE HISTORY AND EXAMINATION

A 61‐year‐old man from the Southern part of Ethiopia, presented with an 8‐month history of nonspecific abdominal pain which later became associated with bilateral leg swelling of 1 month duration. He has no family history of cancer or any known comorbid illness.

Examination revealed an ill‐defined mass over the right side of the lower abdomen with visible anterior abdominal wall collaterals and bilateral lower extremity edema.

## 3 DIFFERENTIAL DIAGNOSIS AND INVESTIGATION

**FIGURE 1:**
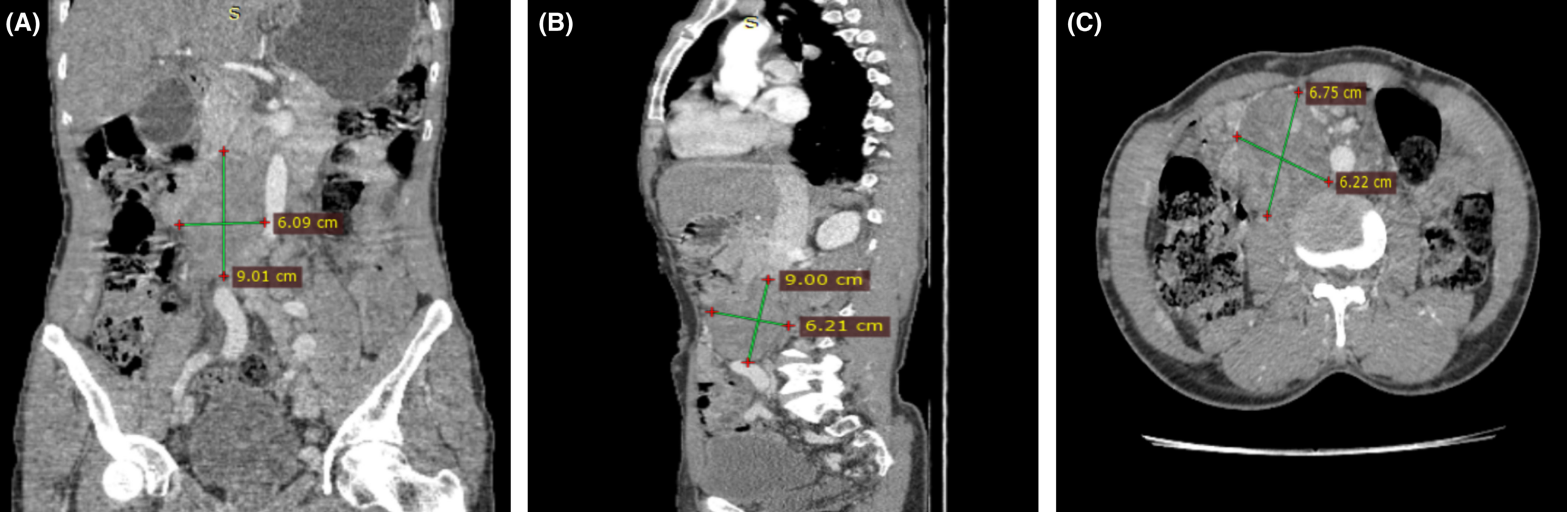
Preoperative Computed Tomography (CT) Angiogram of the Abdomen at images (A–C) (A) Coronal view showing a heterogeneously enhancing mass involving the whole length of the infrarenal inferior vena cava, (B) Sagittal View revealing a 9 × 6.2 cm inferior vena cava mass, (C) Axial View showing a mass obstructing the inferior vena cava lumen.

The top differentials entertained were primary retroperitoneal sarcoma, malignant tumor thrombus, neurogenic tumor, and retroperitoneal teratoma. An abdominal ultrasound showed a large hypoechoic right‐side retroperitoneal mass invading the IVC with a possible differential of malignant intra‐abdominal mass with mass effect on the IVC. Doppler revealed extensive bilateral acute DVT involving femoral, external, internal iliac, and distal IVC. Computed Tomography (CT) Angiogram of the Abdomen revealed a 9 × 6.2 × 6.75 cm heterogeneously enhancing mass involving the whole length of the infrarenal IVC obstructing the IVC lumen with collateral veins draining through the paralumbar veins and inferior epigastric veins bilaterally (Figure 1).

The top differential from the CT scan review and clinical correlation was Primary IVC LMS. Chest CT was unremarkable.

## 4 TREATMENT, OUTCOME, AND FOLLOW‐UP

He was started on warfarin for the diagnosis of bilateral acute deep venous thrombosis (DVT). The medication was then changed to Rivaroxaban. A midline longitudinal laparotomy was performed and the peritoneal cavity was explored. Cattell‐Braasch maneuver was done to mobilize the right side of the colon and small bowel to the left side with protection of the right ureter. The intraoperative finding was a big tumor arising from infra‐renal IVC extending up to the confluence of the common iliac veins. There was no invasion of the aorta, kidneys, and duodenum. There was no suspicious lymph node, liver lesion, peritoneal seeding, or ascites. The confluence of the common iliac vein was dissected underneath the right common iliac artery. There was an injury to the right common iliac artery which later was managed by end‐to‐end anastomosis. Heparin was given and the proximal extent of the tumor was clamped just distal to renal veins and the entire tumor was removed. A 24 mm Gore‐Tex graft was used to reconstruct the IVC. The patient required 04 units of packed Red Blood Cell (RBC) transfusion intra‐operatively. His postoperative course was uneventful.

**FIGURE 2:**
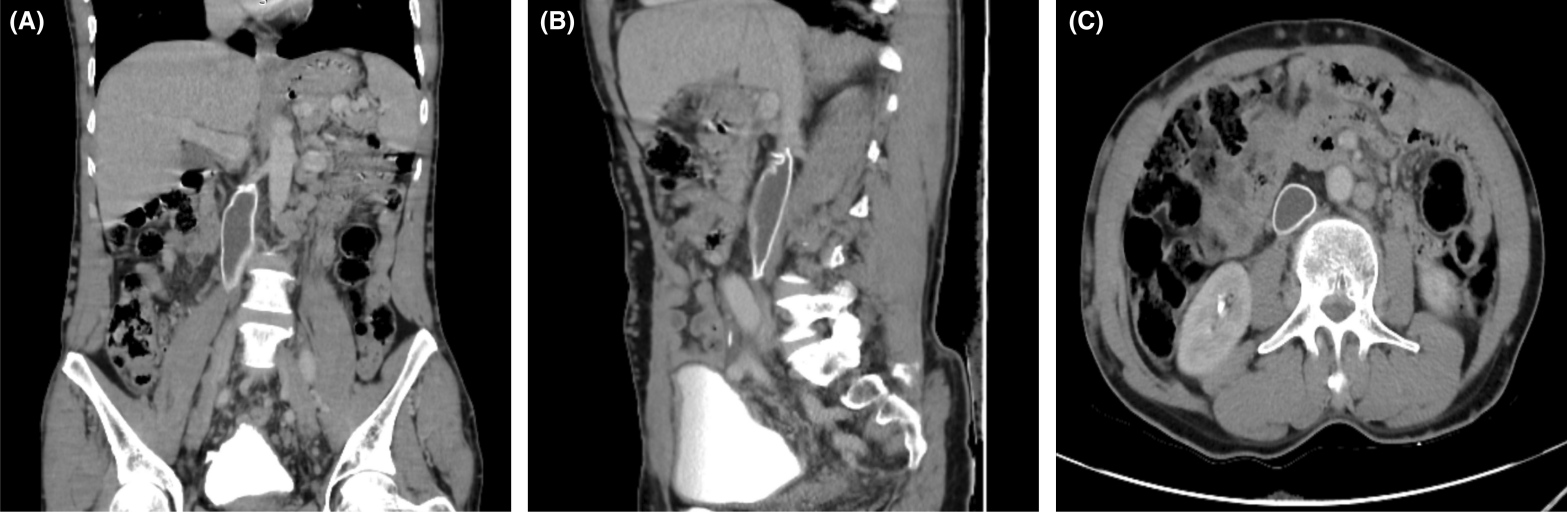
Postoperative abdominal Computed Tomography (CT) Scan at images (A–C) showing a functional surgical graft connecting the proximal inferior vena cava to the left common iliac vein (A, Coronal View; B, Sagittal View; C, Axial View).

Histopathology of the specimen grossly reported an encapsulated gray‐white to gray‐brownish globular mass measuring 12 × 7 × 5 cm. Microcopy shows intersecting fascicles of oval to spindle cells having significant pleomorphism. Eight out of ten mitoses per high power field were seen. The final index was a high‐grade LMS of the IVC. The surgical margin status was unknown as the biopsy was submitted without labeling, and the sample was fragmented during handling and processing. Postoperation Abdominal CT was reported as surgical site loculated fluid collection likely seroma but no gross residual tumor. The surgical implant connecting the proximal IVC to the left common iliac vein was also visualized (Figure 2).

Postoperation Chest CT scan was unremarkable. A combination chemotherapy regimen with Doxorubicin and Dacarbazine every 3 weeks was decided. The patient's performance status with Eastern Cooperative Oncology Group (ECOG) is I. He has taken 4 cycles of Doxorubicin and Dacarbazine. He tolerated the chemotherapy with no Grade 2 or above adverse effects according to the Common Terminology Criteria for Adverse Events (CTCAE) Version 5. Mid‐cycle workup with contrast‐enhanced Abdominal CT and Chest CT showed no evidence of disease. He is on follow‐up at the Oncology side with a plan to complete the chemotherapy up to 6 cycles.

## 5 DISCUSSION

Due to the rarity of this disease, most of the existing literature consists of case reports and case series, making it challenging to establish uniform clinical practices.

A pooled analysis of IVC LMS showed a female predominance of 3:1 with higher incidence observed in the 5th and 6th decade of life.^3^, ^5^ Most IVC LMS grow extraluminally followed by a combined pattern of extraluminal and intraluminal growth and rarely they may grow intraluminally only.^6^, ^7^, ^8^ Depending on their location, they can be classified as Level I (the infra‐renal IVC), level II (the para‐renal, supra‐renal, and infra‐hepatic IVC), and Level III (the supra‐hepatic). The commonest site is Level II.^9^, ^10^, ^11^ Patients with LMS of IVC usually present insidiously with vague abdominal pain, abdominal mass, lower extremity swelling, weight loss, easy fatigability, and, vomiting.^3^, ^5^, ^12^ Around 50% of IVC LMS present with metastasis.^8^

The diagnosis of IVC LMS can be made by contrast‐enhanced CT or Magnetic Resonance Imaging (MRI).^7^, ^13^ However, a definite diagnosis and assessment of resectability is only made through Exploratory laparotomy with surgical biopsy. These tumors have variable anatomical involvement for which different surgical resection techniques and approaches should be tailored according to their characteristics and locations.^6^, ^11^ Surgical access methods included bilateral subcostal or midline abdominal incisions. In our patient's case, a midline laparotomy was utilized due to the tumor location.

When performing IVC surgery, surgeons can choose between primary repair, patch, or IVC reconstruction, depending on the tumor's size and location.^3^, ^6^, ^11^ Literature supports IVC reconstructions as a safe and effective surgical approach to achieve oncologic surgical goals, including clear margins while maintaining patient safety. Given the specific characteristics of our patient's tumor, the decision was made to undergo IVC reconstruction.

Wachtel et al^14^ reported a median disease‐free survival (DFS) of 12 months and a median overall survival (OS) of approximately 23 months. The OS was 92% at 1 year and 55% at 5 years.^14^ Several factors impact DFS and OS, surgical margin status is one of the most important factors predicting outcome.

Two case series highlight the importance of clear surgical margins: Hines et al.^15^ reported a 5‐year survival of 68% in patients with clear margins compared to 0% in patients with involved margins. Hollenbeck et al.^16^ found a similar trend, with a 5‐year DSF of 33% in patients with negative margins versus 0% in patients with positive margins. The histopathology of retroperitoneal soft tissue sarcoma is another predictor of survival. Well‐differentiated tumors tend to have better OS when compared to poorly differentiated tumors.^10^

In adherence to the National Comprehensive Cancer Network (NCCN) treatment guideline for retroperitoneal/intra‐abdominal sarcoma, Doxorubicin‐based adjuvant systemic therapy was initiated for our patient. The guideline states that adjuvant chemotherapy could be considered for patients with a high risk for metastasis as evidenced by R1 or R2 Resection and high grade on histopathology. Adjuvant Radiation is discouraged as it is difficult to define tumor target volume. Additionally, the postoperative bed will be covered by adjacent abdominal organs and bowel loops causing radiation toxicity with no proven benefit in terms of oncologic outcome. A neoadjuvant approach with radiotherapy is favored to make tumors more amenable for resection. Neoadjuvant systemic therapy can be given in highly selected cases to downstage the tumor and decrease the risk of metastatic disease.^17^ In the case described, the decision was made to give combination therapy since the patient had a high‐grade LMS and its surgical margin status was unknown due to a fragmented sample submitted for histopathology.

## 6 CONCLUSION

LMS of the IVC are incredibly rare tumors. To our knowledge, this is the first case report highlighting the diagnostic approach and management of IVC LMS from Ethiopia. Complete surgical resection with a clear margin and a multidisciplinary team evaluation and management are recommended for successful outcomes. The evidence for the utilization of radiation, chemotherapy, or chemoradiation remains uncertain. Providing adjuvant chemotherapy can be beneficial if there is a high risk for metastatic disease.

## AUTHOR CONTRIBUTIONS

**Feleke Hailmariam Maniso:** Conceptualization; data curation; formal analysis; investigation; methodology; validation; visualization; writing – original draft; writing – review and editing. **Mathewos Assefa Woldegeorgis:** Methodology; supervision; validation; visualization; writing – original draft; writing – review and editing. **Hawi Furgassa Bedada:** Formal analysis; investigation; methodology; validation; visualization; writing – original draft; writing – review and editing.

## FUNDING INFORMATION

The study is not funded.

## CONFLICT OF INTEREST STATEMENT

None declared.

## CONSENT

Written informed consent was obtained from the patient to publish this report in accordance with the journal's patient consent policy.

## Data Availability

The data used to support the findings of this case report are available from the corresponding author upon request.

## References

[1] Roland CL , Boland GM , Demicco EG , et al. Primary vascular leiomyosarcoma: clinical observations and molecular variables. JAMA Surg. 2016;151(4):347‐354. 26629783 10.1001/jamasurg.2015.4205 PMC4941943

[2] Burke AP , Virmani R . Sarcomas of the great vessels. A Clinicopathologic Study Cancer. 1993;71(5):1761‐1773. 8448740 10.1002/1097-0142(19930301)71:5<1761::aid-cncr2820710510>3.0.co;2-7

[3] Pan J , Qiu C‐y , He Y‐y , et al. A 10‐year experience of leiomyosarcoma of the inferior vena cava. Phlebology. 2022;37(8):572‐578. 35570826 10.1177/02683555221101706

[4] Teixeira FJR Jr . Leiomyosarcoma of the inferior vena cava: survival rate following radical resection. Oncol Lett. 2017;14(4):3909‐3916. 29098019 10.3892/ol.2017.6706 PMC5651407

[5] Saikia J . A systematic review of the current management approaches in leiomyosarcoma of inferior vena cava‐results from analysis of 118 cases. Asian Cardiovasc Thorac Ann. 2022;30(3):349‐363. 34672808 10.1177/02184923211049911

[6] Dew J , Hansen K , Hammon J , McCoy T , Levine EA , Shen P . Leiomyosarcoma of the inferior vena cava: surgical management and clinical results. Am Surg. 2005;71(6):497‐501. 16044929 10.1177/000313480507100609

[7] Webb EM , Wang ZJ , Westphalen AC , Nakakura EK , Coakley FV , Yeh BM . Can CT features differentiate between inferior vena cava leiomyosarcomas and primary retroperitoneal masses? Am J Roentgenol. 2013;200(1):205‐209. 23255763 10.2214/AJR.11.7476

[8] Mingoli A , Cavallaro A , Sapienza P , Di Marzo L , Feldhaus R , Cavallari N . International registry of inferior vena cava leiomyosarcoma: analysis of a world series on 218 patients. Anticancer Res. 1996;16(5B):3201‐3205. 8920790

[9] Ameeri S , Butany J , Collins MJ , et al. Leiomyosarcoma of the inferior vena cava. Cardiovasc Pathol. 2006;15(3):171‐173. 16697934 10.1016/j.carpath.2005.08.011

[10] Kulaylat MN , Karakousis CP , Doerr RJ , Karamanoukian HL , O'Brien J , Peer R . Leiomyosarcoma of the inferior vena cava: a clinicopathologic review and report of three cases. J Surg Oncol. 1997;65(3):205‐217. 9236931 10.1002/(sici)1096-9098(199707)65:3<205::aid-jso11>3.0.co;2-2

[11] Kieffer E . Leiomyosarcoma of the inferior vena cava: experience in 22 cases. Ann Surg. 2006;244(2):289‐295. 16858193 10.1097/01.sla.0000229964.71743.db PMC1602179

[12] Alkhalili E , Greenbaum A , Langsfeld M , et al. Leiomyosarcoma of the inferior vena cava: a case series and review of the literature. Ann Vasc Surg. 2016;33:245‐251. 26802297 10.1016/j.avsg.2015.10.016

[13] Mastoraki A , Leotsakos G , Mastoraki S , et al. Challenging diagnostic and therapeutic modalities for leiomyosarcoma of inferior vena cava. Int J Surg. 2015;13:92‐95. 25489949 10.1016/j.ijsu.2014.11.051

[14] Wachtel H . Outcomes after resection of leiomyosarcomas of the inferior vena cava: a pooled data analysis of 377 cases. Surg Oncol. 2015;24(1):21‐27. 25433957 10.1016/j.suronc.2014.10.007

[15] Hines OJ . Leiomyosarcoma of the inferior vena cava: prognosis and comparison with leiomyosarcoma of other anatomic sites. Cancer. 1999;85:5. 10091791

[16] Hollenbeck ST . Surgical treatment and outcomes of patients with primary inferior vena cava leiomyosarcoma. J Vasc Surg Venous Lymphat Disord. 2003;197:4. 10.1016/S1072-7515(03)00433-2 14522326

[17] National Comprehensive Cancer Network (NCCN) guidelines . Soft Tissue Sarcoma. 2024 Available from: https://www.nccn.org/guidelines/guidelines‐detail?category=1&id=1464

